# Antimicrobial Activity of Sphingolipids Isolated from the Stems of Cucumber (*Cucumis sativus* L.)

**DOI:** 10.3390/molecules15129288

**Published:** 2010-12-15

**Authors:** Jing Tang, Xiangjie Meng, Hao Liu, Jianglin Zhao, Ligang Zhou, Minghua Qiu, Xianming Zhang, Zhu Yu, Fuyu Yang

**Affiliations:** 1 College of Agronomy and Biotechnology, China Agricultural University, Beijing 100193, China; 2 State Key Laboratory of Phytochemistry and Plant Resources in West China, Kunming Institute of Botany, Chinese Academy of Sciences, Kunming 650204, China; 3 College of Animal Science and Technology, China Agricultural University, Beijing 100193, China

**Keywords:** Cucurbitaceae, cucumber (*Cucumis sativus* L.), sphingolipid, antimicrobial activity, phytopathogenic fungi and bacteria

## Abstract

Three antimicrobial sphingolipids were separated by bioassay-guided isolation from the chloroform fraction of the crude methanol extract of cucumber (*Cucumis sativus* L.) stems and identified as (*2S,3S,4R,10E*)*-*2-[(2'*R*)-2-hydroxytetra-cosanoylamino]-1,3,4-octadecanetriol-10-ene (**1**), 1-*O*-*β*-D-glucopyranosyl(*2S,3S,4R,10E*)-2-[(2'*R*)-2-hydroxy-tetracosanoylamino]-1,3,4-octadecanetriol-10-ene (**2**) and soya-cerebroside I (**3**) by their physicochemical properties and spectroscopic analysis. They were evaluated to show antifungal and antibacterial activity on test microorganisms including four fungal and three bacterial species. Among them, compound **1**, a relatively low polarity aglycone, exhibited stronger antimicrobial activity than its corresponding glycoside **2**. The results indicated that sphingolipids could be the main antimicrobial compounds in the crude methanol extract of cucumber stems.

## 1. Introduction

Plants are capable of synthesizing a diverse array of secondary metabolites. These may be produced constitutively (preformed antimicrobial compounds, or phytoanticipins) or in response to pathogen or herbivore attack or stress (phytoalexins) [1]. There has been renewed interest over the last 20 years in the isolation of antimicrobial compounds from plants because of their structural diversity, unique bioactivity and environmental compatibility, which are more favorable than those of synthetic chemicals [2,3].

Cucumber (*Cucumis sativus* L.), which belongs to the Cucurbitaceae, is now widely planted in the temperate and tropical zones, including all the districts in China [4]. It is one of the most important vegetables, and the stems have been used in Traditional Chinese Medicine for their anti-inflammatory activity. According to the ancient book "Ben Cao Gang Mu" edited by Shizhen Li of the Ming Dynasty of China, the stems can expand the blood vessels and reduce blood pressure [5]. However, very little is known about the antimicrobial constituents from cucumber stems, though some reports have suggested the presence of steroids and phenolics in this plant [6,7]. The present study aimed to isolate and identify the antimicrobial sphingolipids from cucumber stems based on bioassay-guided fractionation.

## 2. Results and Discussion

### 2.1. Elucidation of the purified sphingolipids

**Figure 1:**
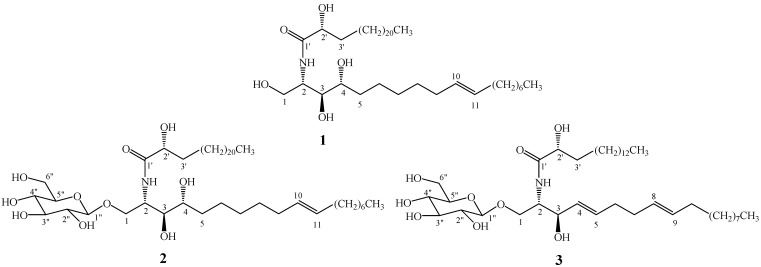
Chemical structures of the compounds **1**-**3**.

Three sphingolipids were isolated from the chloroform fraction of the crude methanol extract from cucumber stems based on bioassay-guided fractionation. After comparing their physicochemical and spectrometric data with those reported in the literature [8,9,10,11,12,13], they were identified as known compounds and confirmed as (*2S,3S,4R,10E*)*-*2-[(2'*R*)-2-hydroxytetracosanoylamino]-1,3,4-octa- decanetriol-10-ene (**1**), 1-*O*-*β*-D-glucopyranosyl-(*2S,3S,4R,10E*)-2-[(2'*R*)-2-hydroxytetracosan-oylamino]-1,3,4-octadecanetriol-10-ene (**2**) and soya-cerebroside I (**3**), whose structures were shown in Figure 1.

(*2S,3S,4R,10E*)*-*2-[(2'*R*)-2-Hydroxytetracosanoylamino]-1,3,4-octadecanetriol-10-ene (**1**) was first isolated from the medicinal fungus *Engleromyces goetzei* [8]. 1-*O*-*β*-D-Glucopyranosyl-(*2S,3S, 4R,10E*)-2-[(2'*R*)-2-hydroxytetracosanoylamino]-1,3,4-octadecanetriol-10-ene (**2**) was first isolated from the roots of *Incarvillea arguta* (Bignoniaceae) [9]. Soya-cerebroside I (1-*O*-*β*-D-glucopyranosyl-(2S,3R,4*E*,8*E*)-2-*N*-2'-hydroxypalmitoyl-4,8-sphingadienine, **3**) was first isolated from *Tetragonia tetragonoides* (Aizoaceae) and named cerebroside B1a [10], later from the seeds of *Glycine max* (Leguminosae) [11], the aerial parts of *Cinnamomun zeylanicum* (Lauraceae) [12], and whole plant of *Zehneria maysorensis* (Cucurbitaceae) [13]. Fang *et al*. [14] reported that soya-cerebroside I (**3**) content in pumpkin (*Cucurbita pepo*) seeds was 0.031 mg/g according to a single quadruple liquid chromatography-mass spectrometry (LC-MS) method. These sphingolipids are widely distributed in plants and fungi.

### 2.2. Antimicrobial activity

Mycelial growth inhibitory activity of the crude methanol extract and its different polar fractions of cucumber stems against fungal growth was summarized in Table 1. Both chloroform fraction and *n*-butanol fraction exhibited stronger inhibitory activity than methanol extract and aqueous fraction, though the activity of chloroform and *n*-butanol fractions was weaker than that of the positive control (carbenzazim).

**Table 1:** Mycelia growth inhibitory activity of the crude methanol extract and its fractions of cucumberstems against four phytopathogenic fungi.

Treatment	Inhibitory rate of the mycelia growth (mean ± SD) (%)			
	*P. aphanidermatum*	*B. dothidea*	*B. cinerea*	*F. oxysporum* f.sp. *cucumerinum*
Carbendazim	79.2 ± 2.0	88.0 ± 0.6	29.0 ± 2.0	100.0 ± 0.0
Methanol extract	45.0 ± 2.4	7.6 ± 3.3	20.4 ± 1.4	35.0 ± 3.5
Chloroform fraction	49.4 ± 0.9	41.3 ± 1.0	44.8 ± 1.7	42.2 ± 1.6
*n*-Butanol fraction	48.5 ± 2.0	16.5 ± 3.5	41.4 ± 2.8	41.6 ± 1.3
Aqueous fraction	43.8 ± 1.6	6.8 ± 1.8	15.6 ± 2.4	23.4 ± 2.7

Note: Positive control was carbendazim (10 μg/mL). Negative control was ethanol which was tested to have no inhibitory activity. Concentration of the extract or fraction in medium was 1.0 mg/mL.

Inhibitory activity of the crude methanol extract and its fractions on bacterial growth is summarized in Table 2. Except for the aqueous fraction, the others exhibited moderate antibacterial activity on the three tested bacteria. Among them, the chloroform fraction exhibited the strongest inhibitory activity against the three tested bacteria.

Results by two different bioassays (shown in Table 1 and Table 2) indicated that the chloroform fraction had stronger antimicrobial activity than the other ones. Therefore, further purification of antimicrobial compounds by column chromatography was focused on the chloroform fraction.

**Table 2:** Bacteria growth inhibition activity of the crude methanol extract and fractions of cucumber stems.

Treatment	Diameter of inhibitory zone (mm)		
	*B. subtilis*	*X. vesicatoria*	*P. lachrymans*
Streptomycin sulfate	+++	+++	+++
Methanol extract	+	+	++
Chloroform fraction	++	++	+++
*n*-Butanol fraction	+	+	+
Aqueous fraction	-	-	-

Note: The quantity of the extract or fractions in each well was 2.5 mg, and that of streptomycin sulfate in each well was 8 μg. The solvent was DMSO which was tested to have no inhibitory activity. "-", no inhibitory zone; "+", 0< D ≤5mm; "++", 5< D ≤10mm; "+++", D > 10mm.

Three sphingolipids were screened to have strong antifungal activity on *Pythium aphanidermatum* and *Botrytis cinerea* with the results being reported in Table 3. It showed that mycelial growth inhibitory rates of the three compounds against *B. cinerea* at a concentration of 100 μg/mL in medium were higher than or close to those of carbendazim (10 μg/mL in medium). Their inhibitory rates on *P. aphanidermatum* were 100.0%, 46.6% and 23.5%, respectively. Compounds **1**, **2 **and**3** were also screened to have their strong antibacterial activity against the three tested bacteria, and the IC_50_ data were reported in Table 4. All three compounds exhibited the strongest inhibitory activity on *Pseudomonas lachrymans* with IC_50_ values being 15.3 μg/mL, 17.4 μg/mL and 37.3 μg/mL, respectively.

**Table 3:** Mycelia growth inhibition of the compounds **1**-**3** against four phytopathogenic fungi.

Treatment	Inhibitory rate of the mycelia growth (Mean ± SD) (%)			
	*P. aphanidermatum*	*B. dothidea*	*B. cinerea*	*F. oxysporum* f.sp. *cucumerinum*
Compd 1	100.0 ± 0.0	22.3 ± 2.4	48.4 ± 1.0	10.7 ± 2.9
Compd 2	46.6 ± 2.5	11.2 ± 3.1	30.4 ± 1.8	9.3 ± 1.5
Compd 3	23.5 ± 4.3	7.1 ± 3.2	24.4 ± 2.2	5.5 ± 1.0

Note: Both the positive and negative controls were the same as those in Table 1. Concentration of each compound in medium was 100 μg/mL.

**Table 4:** Median effective inhibitory concentration (IC_50_) of compounds **1**-**3** on phyto-pathogenic bacteria growth.

Compound	Test bacterium	Toxicity regression equation (*Y* = a*X* + b)	Correlation coefficient (*R*)	IC_50_(μg/mL)
Compd 1	*B. subtilis*	*Y* = 0.8321*X* + 3.5848	0.9793	50.2
	*X. vesicatoria*	*Y* = 0.8918*X* + 3.7441	0.9864	25.6
	*P. lachrymans*	*Y* = 0.3952*X* + 4.5318	0.9752	15.3
Compd 2	*B. subtilis*	*Y* = 0.9746*X* + 3.4555	0.9893	87.9
	*X. vesicatoria*	*Y* = 0.9202*X* + 3.6106	0.9818	32.4
	*P. lachrymans*	*Y* = 0.3844*X* + 4.5234	0.9841	17.4
Compd 3	*B. subtilis*	*Y* = 0.8842*X* + 3.1918	0.9398	110.9
	*X. vesicatoria*	*Y* = 0.9775*X* + 3.2311	0.9886	64.5
	*P. lachrymans*	*Y* = 0.6110*X* + 4.0395	0.9712	37.3

Note: Toxicity regression equation *Y* = a*X* + b, where *Y* is the inhibitory probit value, and *X* is concentration logarithm of compounds. The IC_50_ values of the positive control streptomycin sulfate on *B. subtilis*, *X. vesicatoria* and *P. lachrymans* were 5.0 μg/mL, 11.6 μg/mL and 9.0 μg/mL, respectively.

Of the three sphingolipids, compound **1** was the aglycone of compound **2**, and compound **3** was another sphingolipid glycoside. Compound **1** was screened to show strong antibacterial and antifungal activity. Both two glycosides (*i.e*., **2** and **3**) exhibited relatively weak antimicrobial activity. Except sfor oya-cerebroside I (**3**) which was shown to have protective activity against ulcer formation in mice under restraint and water immersion conditions [10], the other two sphingolipids (**1** and **2**) have not been previously reported to possess biological activity. Sphingolipids have been found widely in plants, animals and fungi as components of biomembranes. They are of special interest because of their physiological role in the signaling pathway [15]. The role of these sphingolipids in cucumber needs to be studied in more detail.

## 3. Experimental

### 3.1. General

The melting points were determined on an XRC-1 micro-melting point apparatus and are uncorrected. NMR spectra (^1^H-NMR, ^13^C-NMR and DEPT) were recorded on either a Varian Bruker AV-400 spectrometer at 100 MHz for ^13^C or a DRX-500 NMR spectrometer at 500 MHz for ^1^H. The chemical shifts were expressed in ppm as *δ* values relative to tetramethylsilane (TMS) as an internal standard. MS spectra were recorded on VG Auto Spec-3000 mass spectrometer. Column chromatography was performed on either silica gel (200-300 mesh, Qingdao Marine Chemical Company, China) or Sephadex LH-20 (25-100 μm, Pharmacia Company). TLC was performed on pre-coated silica gel F_254_ plates (Qingdao Marine Chemical Company, China). Detection was provided by UV at 254 nm, spraying with 10% H_2_SO_4_-EtOH followed by heating at 100 °C. A microplate spectrophotometer (PowerWave HT, BioTek Instruments, USA) was employed to measure the light absorption value. 3-(4,5-Dimethylthiazol-2-yl)-2,5-diphenyl tetrazolium bromide (MTT) was purchased from Amresco (USA). All other chemicals and reagents were of analytical grade.

### 3.2. Plant material

The stems of cucumber (*Cucumis sativus* L.) were collected in August 2008 in Lijiang of Yunnan Province in the southwest of China, and plant specimen was identified by Prof. Shukun Chen of Kunming Institute of Botany. The stems were left to dry in the shade at room temperature to a constant weight. A voucher specimen was deposited in the Herbarium of the Department of Taxonomy, Kunming Institute of Botany, Chinese Academy of Sciences.

### 3.3. Extraction, fractionation and identification of the sphingolipids

The air-dried cucumber stems (4.5 kg) were ground into powder and then extracted three times with 20 L of methanol under reflux at 60 °C, for four hours each time. The combined filtrate was concentrated *in vacuo* at 50 °C using a rotary evaporator to afford a crude methanol extract residue (330 g), which was further suspended in 2 L of water and extracted successively with 2 L of chloroform and then with 2 L of *n*-butanol to give a chloroform fraction (65 g), and a *n*-butanol fraction (60 g) after concentration. The remaining water layer was concentrated as the aqueous fraction (170 g). The fractions were stored in a refrigerator at 4 °C before used (for the antimicrobial activity test or further fractionation). The chloroform fraction (60 g) was subjected to column chromatography on a silica gel (1,200 g, 200-300 mesh), eluting with the gradient mixture of CHCl_3_-MeOH (from 50:1 to 5:1, v/v) to yield five sub-fractions based on TLC analysis. Among them, sub-fractions 3 and 5 showed antimicrobial activity and were further separated. Sub-fraction 3 (3.5 g) was repeatedly chromatographed over silica gel (200-300 mesh) eluted with CHCl_3_-MeOH (from 30:1 to 10:1, v/v), and on Sephadex LH-20 eluting with MeOH to yield compound **1** (146 mg). Compounds **2** (674 mg) and **3** (164 mg) were obtained from sub-fraction 5 (13.0 g) by repeated silica gel chromatography using CHCl_3_-MeOH (from 10:1 to 5:1, v/v) as solvent system. The physicochemical and spectrometric data of three sphingolipids were given as follows.

*(2S,3S,4R,10E)-2-[(2'R)-2-hydroxytetracosanoylamino]-1,3,4-octadecanetriol-10-ene* (**1**). White amorphous powder (MeOH); m.p. 138-139 °C; C_42_H_83_NO_5_; FAB-MS (negative) *m/z*, 680 ([M-H]^-^); ^1^H-NMR (pyridine-*d_5_*) *δ* (ppm), 8.57 (1H, d, *J* = 8.8 Hz, NH), 5.52 (1H, m, H-10), 5.50 (1H, m, H-11), 5.10 (1H, d, *J* = 4.3 Hz, H-2), 4.61 (1H, m, H-2'), 4.51 (1H, brs, H-1), 4.42 (1H, m, H-1), 4.33 (1H, m, H-3), 4.27 (1H, m, H-4), 1.25-1.30 (54H, m, 27×CH_2_), 0.85 (6H, t-like, *J* = 6.7 Hz, Me-18 and Me-24'); ^13^C-NMR (pyridine-*d_5_*) *δ* (ppm), 62.0 (C-1), 52.9 (C-2), 76.9 (C-3), 73.0 (C-4), 33.3 (C-5), 26.8 (C-6), 32.2 (C-9), 130.8 (C-10), 130.7 (C-11), 33.0 (C-12), 175.3 (C-1'), 72.5 (C-2'), 35.7 (C-3'), 25.8 (C-4'), 29.6-30.3 (C-13-16 and C-5'-22'), 22.9 (C-17 and C-23'), 14.3 (C-18 and C-24'). The structure was confirmed by comparison with literature data [8].

*1-O-β-D-glucopyranosyl-(2S,3S,4R,10E)-2-[(2'R)-2-hydroxytetracosanoylamino]-1,3,4-octadecane-triol-10-ene* (**2**). White amorphous powder (MeOH); m.p. 203-205 °C; C_48_H_93_NO_10_; FAB-MS (negative) *m/z*, 842 [M-H]^-^, 680; ^1^H-NMR (pyridine-*d_5_*) *δ* (ppm), 8.54 (1H, d, *J* = 8.8 Hz, NH), 5.50 (1H, m, H-10), 5.49 (1H, m, H-11), 5.27 (1H, d, *J* = 4.3 Hz, H-2), 4.93 (1H, d, *J* = 6.9 Hz, H-1"), 1.24-1.31 (54H, m, 27×CH_2_), 0.85 (6H, t-like, *J* = 6.7 Hz, Me-18 and Me-24'); ^13^C-NMR (pyridine-*d_5_*) *δ* (ppm), 70.4 (C-1), 51.9 (C-2), 75.9 (C-3), 72.5 (C-4), 33.9 (C-5), 33.0 (C-6), 32.9 (C-7), 32.8 (C-8), 32.2 (C-9), 130.9 (C-10), 130.7 (C-11), 33.3 (C-12), 175.7 (C-1'), 72.5 (C-2'), 35.6 (C-3'), 25.9 (C-4'), 29.6-30.0 (C-13-16 and C-5'-22'), 23.0 (C-17 and C-23'), 14.3 (C-18 and C-24'); Glc: 105.5 (C-1"), 75.2 (C-2"), 78.5 (C-3"), 71.6 (C-4"), 78.6 (C-5"), 62.7 (C-6"). The structure was confirmed by comparison with literature data [9].

*Soya-cerebroside I* (**3**). White amorphous powder (MeOH); m.p. 194-195 °C; C_40_H_75_NO_9_; FAB-MS (negative) *m/z*, 712 [M-H]^-^, 550; ^1^H-NMR (pyridine-*d_5_*) *δ* (ppm), 8.38 (1H, d, *J* = 8.8 Hz, NH), 5.77 (1H, m, H-5), 5.49 (3H, m, H-4, H-8 and H-9), 4.92 (1H, d, *J* = 7.7 Hz, H-1"), 4.51 (1H, m, H-6"b), 4.38 (1H, m, H-6"a), 4.25 (2H, m, H-1a and H-3"), 4.21 (1H, m, H-4"), 4.05 (4H, m, H-1b, H-2, H-3 and H-2"), 3.92 (2H, m, H-2' and H-5"), 2.14 (4H, brs, H-6, H-7), 1.99 (2H, m, H-10), 1.71 (1H, m, H-3'), 1.37 (1H, m, H-4'), 1.25-1.35 (38H, m, 19×CH_2_), 0.86 (6H, t-like, *J* = 6.9 Hz, H-18 and H-16'); ^13^C-NMR (pyridine-*d_5_*) *δ* (ppm), 70.2 (C-1), 54.6 (C-2), 71.5 (C-3), 131.1 (C-4), 132.1 (C-5), 32.2 (C-6), 32.1 (C-7), 130.0 (C-8), 132.1 (C-9), 32.9 (C-10), 175.7 (C-1'), 72.3 (C-2'), 35.7 (C-3'), 25.9 (C-4'), 29.6-30.0 (C-11-16 and C-5'-14'), 23.0 (C-17 and C-15'), 14.3 (C-18 and C-16'); Glc: 105.7 (C-1″), 75.2 (C-2″), 78.5 (C-3″), 71.5 (C-4″), 78.6 (C-5″), 62.7 (C-6″). The structure was confirmed by comparison with literature data [11,12,13].

### 3.4. Antimicrobial activity

#### 3.4.1. Antifungal activity assay

Four phytopathogenic fungal species included *Pythium aphanidermatum*, *Botryosphaeria dothidea*, *Fusarium oxysporum* f.sp. *cucumerinum* and *Botrytis cinerea*, which were supplied by the Department of Plant Pathology of China Agricultural University. *P*. *aphanidermatum* is a pathogen of cucumber damping-off, *B. dothidea* is a pathogen of poplar stem blister canker, *F*. *oxysporum* f.sp. *cucumerinum* is a pathogen of cucumber fungal wilt, and *B*. *cinerea* is a pathogen of tomato gray mold rot. The samples of fungi necessary for the experiments *in vitro* were taken from the cultures grown in slants and kept at 25 ± 1 ºC on potato dextrose agar (PDA).

Mycelial radial growth inhibition assay was used to evaluate antifungal activity of the extract and fractions [16,17]. Briefly, the extract or fraction (100 mg) was dissolved in ethanol (1 mL) and poured into the assay flask containing hot sterilized PDA medium (100 mL) at 50 ºC. After it was thoroughly mixed, about 15 mL of the treated medium was poured into a Petri dish (Ф = 90 mm). The final concentration of the tested sample in medium was 1.0 mg/mL. Control plates were treated with ethanol with a concentration in medium being 1% (v/v). The positive control was carbendazim with a concentration in medium being 10 μg/mL. The assay was performed by placing a 5-mm diameter plug of growing mycelia onto the centre of each treated PDA plate. The radial growth of mycelia in the plates was measured after 3-days inoculation for *P. aphanidermatum* and *B. cinerea*, 5-days inoculation for *B. dothidea* and *F. oxysporum* f.sp *. cucumerinum*. The average was taken of the three measurements made on each Petri dish. Three replicates were used for all treatments. The percentage of mycelial growth inhibition was calculated from mean values using the formula [(*D_c_*-*D_t_*)/*D_c_*] × 100, where *D_c_* is the average diameter increase of fungal colony with the negative control, and *D_t_* is the average diameter increase of a fungal colony with the treatment. To evaluate antifungal activity of the three sphingolipids, the test method was the same as that described above, only the concentration of the compound in medium was different.

#### 3.4.2. Antibacterial activity assay

The bacterial species included two Gram-negative bacteria, *Xanthomonas vesicatoria* ATCC 11633, *Pseudomonas lachrymans* ATCC 11921 and a Gram-positive bacterium, *Bacillus subtilis* ATCC 11562. *X. vesicatoria* is a pathogen of tomato bacterial scab, *P. lachrymans* is a pathogen of cucumber bacterial spot and *B. subtilis* is widely distributed in plants. All bacterial species were obtained from the microbial culture stock in the Department of Plant Pathology, China Agricultural University and maintained in LB medium at 28 ± 1 °C for antibacterial tests.

To evaluate antibacterial activity of the extract or fractions from the plant material, agar-well diffusion assay was employed [18]. Briefly, all components to be tested were dissolved in dimethyl sulfoxide (DMSO) to a final concentration of 50 mg/mL. Hot water agar (15 mL, 3%, w/v) was poured into a Petri-dish (Φ = 90 mm) till solidification, then the bacterial suspension cultured overnight in LB liquid medium (1 × 10^6 ^CFU/mL, 150 µL) was thoroughly mixed with LB agar medium (15 mL) at 50 ºC and was immediately poured onto the solidified water agar layer. The inoculated Petri-dishes were placed uncovered in an incubator at 28 ± 1 °C for 20 min to evaporate the liquid medium on the surface completely. Equidistant wells of 5 mm in diameter were then poured into the solid medium with a sterile cork borer and each was filled with the test solutions (50 µL) at the desired concentration (50 mg/mL). The positive control was streptomycin sulfate with quantity in the well being 8 µg. The culture dishes were kept at 4 ºC for 12 h and then incubated for 24 h at 28 ± 1 °C. The diameter (D, mm) of bacteria-free zone surrounding each well remaining after the incubation period was measured as the antibacterial activity.

To further evaluate the median effective inhibitory concentration (IC_50_) of the three sphingolipids against bacteria, a colorimetric assay by using chromogenic reagent 3-(4,5-dimethylthiazol-2-yl)-2,5-diphenyl tetrazolium bromide (MTT) was employed [19,20]. Briefly, the bacterial suspension cultured overnight in the LB liquid medium (1 × 10^6 ^CFU/mL, 90 μL) was added into a 96-well micro culture plate, then each compound solution (10 μL) was added with their different concentrations. After the culture plate was incubated for 24 h at 28 °C, 10 μL of MTT stock solution at a concentration of 5 mg/mL was added to each well and then the bacteria were incubated for another 4 h at 28 ºC, and the reaction was stopped by adding 100 μL of DMSO. After incubation for 30 min with slight shaking on a microplate shaker at 28 ºC, the plate was centrifuged for 30 min at 1500 g, and then 100 μL of the supernatant (DMSO solution) in each well was transferred to a corresponding well of another 96-well microplate to measure their light absorption values at wavelength 510 nm using a microplate spectrophotometer. The percentage (%) of the bacterial growth inhibition was determined as [(*A*_c_–*A*_t_)/*A*_c_] × 100, where *A*_c_ was an average of six replicates of light absorption values at wavelength 510 nm of the negative controls, and *A*_t_ was an average of six replicates of light absorption values at wavelength 510 nm of the samples. The IC_50_ value was calculated using the linear relation between the inhibitory probability and concentration logarithm according to the method of Sakuma [21].

## 4. Conclusions

In this work, we reported for the first time the isolation of three antimicrobial sphingolipids from cucumber stems by bioassay-guided fractionation. These compounds mainly existed in the chloroform fraction of the crude methanol extract with medium polarity, and were identified as (*2S,3S,4R,10E*)*-*2-[(2'*R*)-2-hydroxytetracosanoylamino]-1,3,4-octadecanetriol-10-ene (**1**), 1-*O*-*β*-D-glucopyranosyl-(*2S, 3S,4R,10E*)-2-[(2'*R*)-2-hydroxytetracosanoylamino]-1,3,4-octadecanetriol-10-ene (**2**) and soya-cerebroside I (**3**). They may be contributors to the antimicrobial activity of the extract and fractions. In addition to the chloroform fraction, the *n*-butanol fraction also showed consistent antimicrobial activity, in some cases being similar to that of the chloroform fraction. It may contain other antimicrobial compounds that deserve isolation and examination of their bioactivity. The results from the present study provided the chemical basis for the efficacy of cucumber stems against plant bacterial and fungal pathogens including those affecting cucumber itself.
